# Supplementary figures and images for: Repeat modules and N-linked glycans define structure and antigenicity of a critical enterotoxigenic E. coli adhesin
Source: PLoS Pathog. 2024 Sep 16;20(9):e1012241. doi: 10.1371/journal.ppat.1012241 (PMC11463764; doi:10.1371/journal.ppat.1012241)

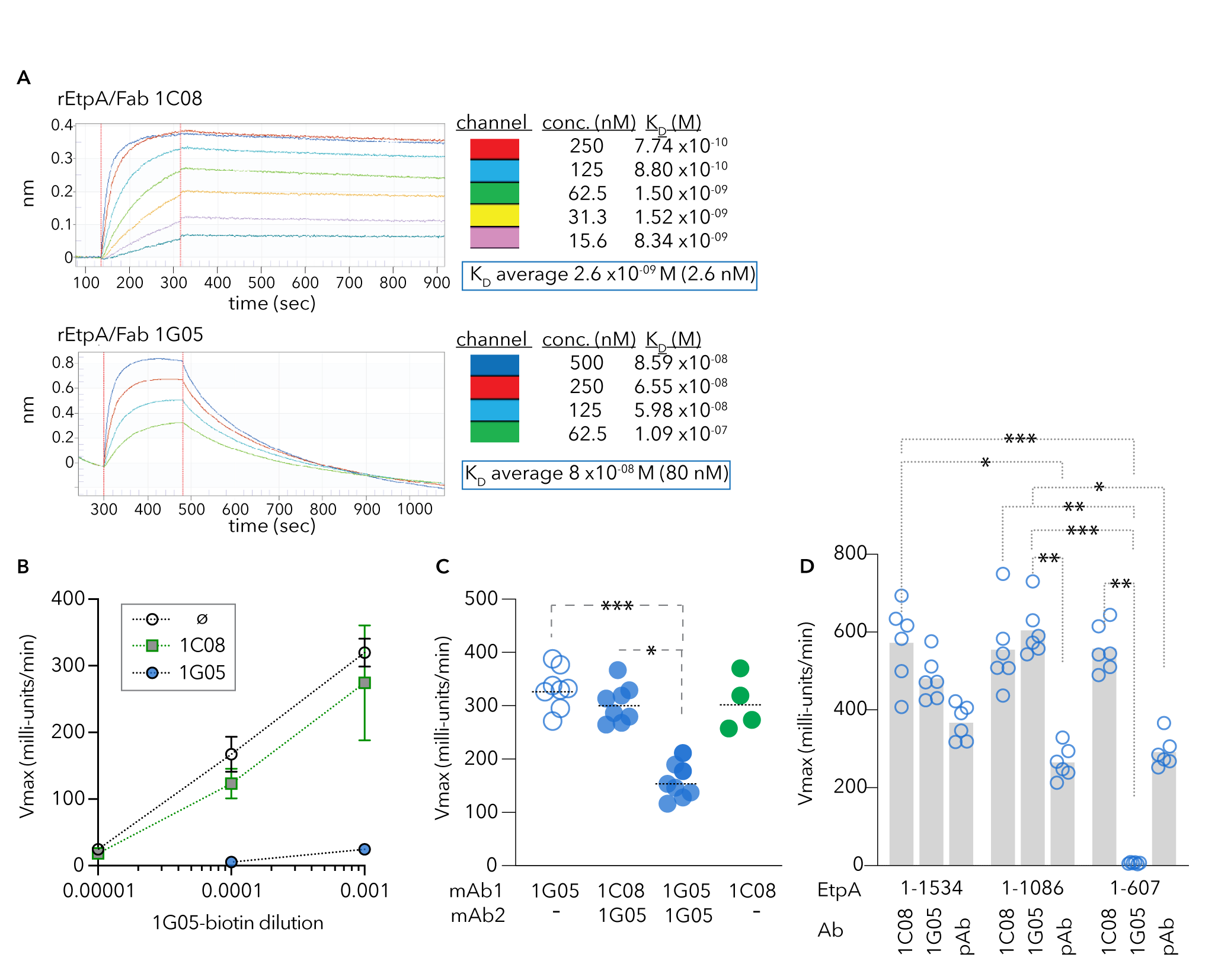

Supplement: S1 Fig — A. Biolayer inferometry (Octet) studies 1C08 and 1G05 Fabs binding to rEtpA. B. mAb 1C08 does not compete for binding with 1G05. Shown are kinetic ELISA data indicating binding of bioinylated 1G05 mAb in the presence of unlabeled 1G05 (blue), 1C08 (green) or alone (open circle). C. 1G05 and 1C08 recognize EtpA but compete for different binding sites. D. 1G05 recognizes the repeat region of EtpA while 1C08 binds the N-terminal secretion domain. Data include n = 6 technical replicates and are representative of three independent experiments. pAb = polyclonal anti-EtpA antibody. Comparisons by Kruskal-Wallis ***≤0.001, **≤0.01, *<0.05. (TIF) [file ppat.1012241.s001.tif]

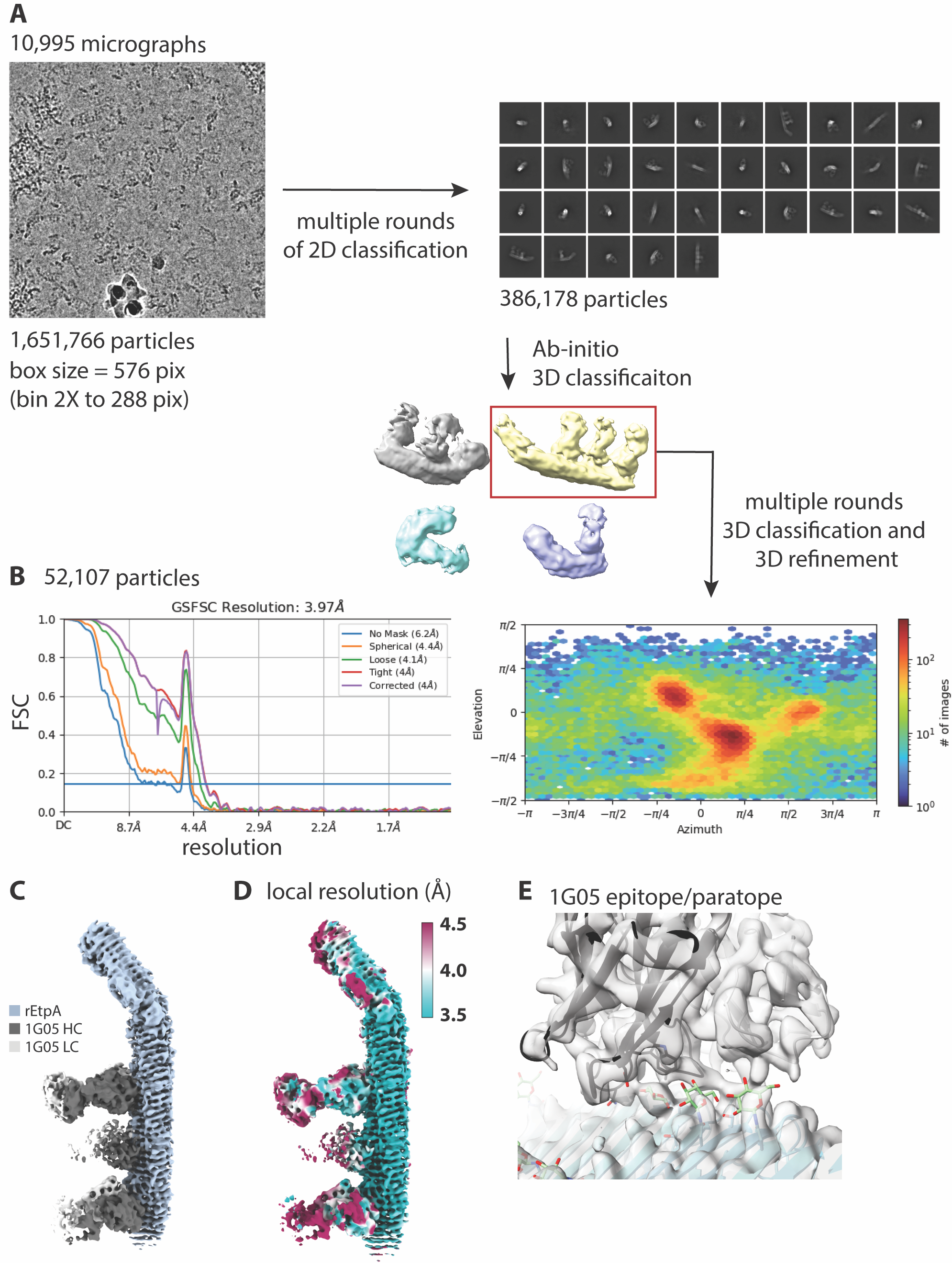

Supplement: S2 Fig — Simplified cryo-EM data processing workflow including A representative example of an aligned and dose-weighted micrograph (lowpass filtered to 5Å), 2D and 3D class averages, and particle counts at each step. B. Angular distribution and Fourier shell correlation plots for the final 3D reconstruction along with the final particle count. C. Sharpened map colored by domain. D. Map colored by local resolution estimate. E. View of the map-model fit in the epitope/paratope region. (TIF) [file ppat.1012241.s002.tif]

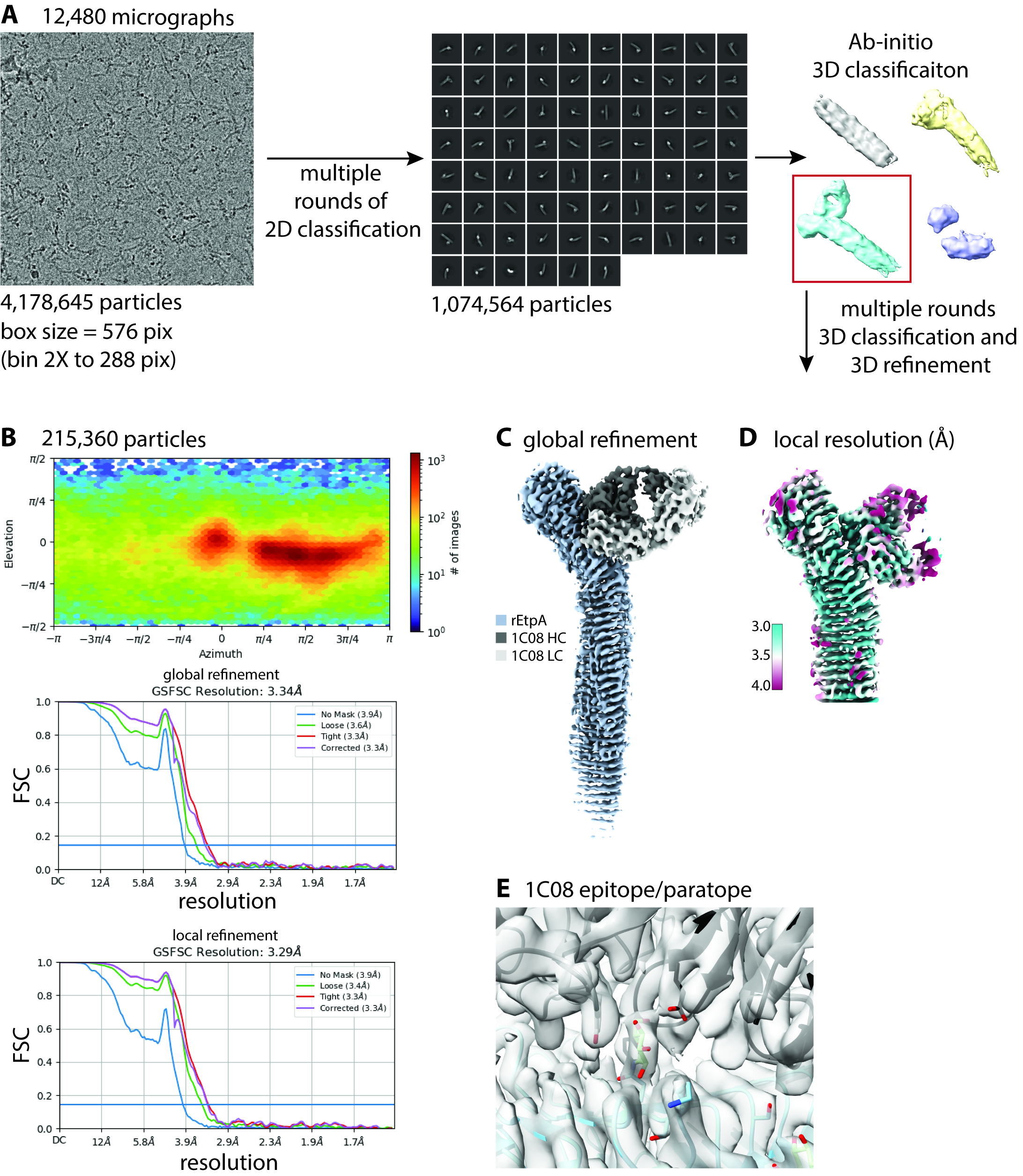

Supplement: S3 Fig — Simplified cryo-EM data processing workflow including representative (A) aligned and dose-weighted micrograph (lowpass filtered to 5Å), 2D and 3D class averages, and particle counts at each step. B. Angular distribution and Fourier shell correlation plots for the final 3D reconstruction along with the final particle count. C. Sharpened map colored by domain. D. Map colored by local resolution estimate. E. View of the map-model fit in the epitope/paratope region. (TIF) [file ppat.1012241.s003.tif]

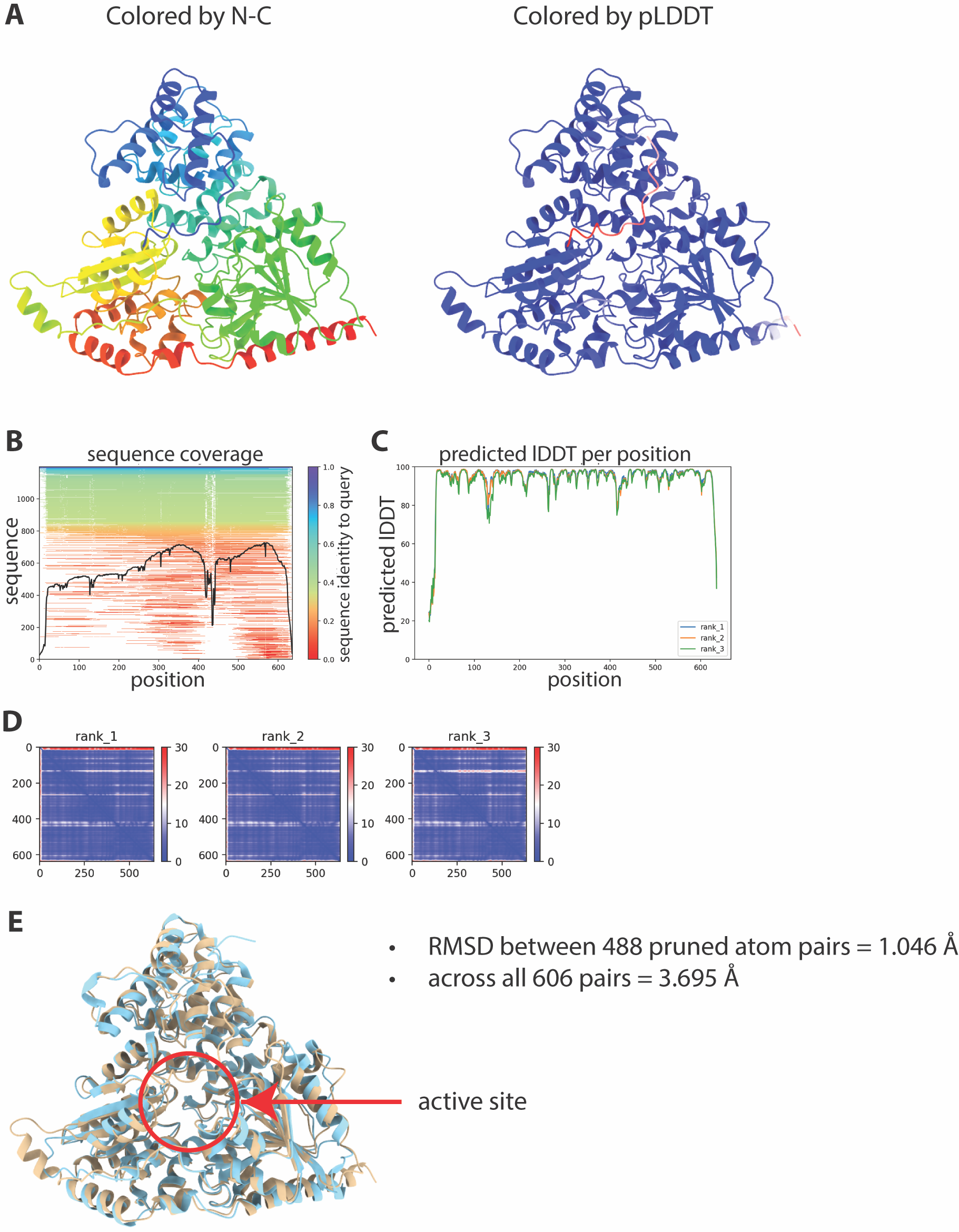

Supplement: S4 Fig — A. Predicted structure of EtpC colored with a rainbow color mapping from the N-terminus to C-terminus and (B) by prediction LDDT confidence store. C. Multiple sequence alignment coverage. D. Predicted LDDT score by residue position. E. Predicted alignment error matricies. F. Structure-based alignment of predicted EtpC structure with the crystal structure of HMW1C (PDBID:3Q3E). (TIF) [file ppat.1012241.s004.tif]

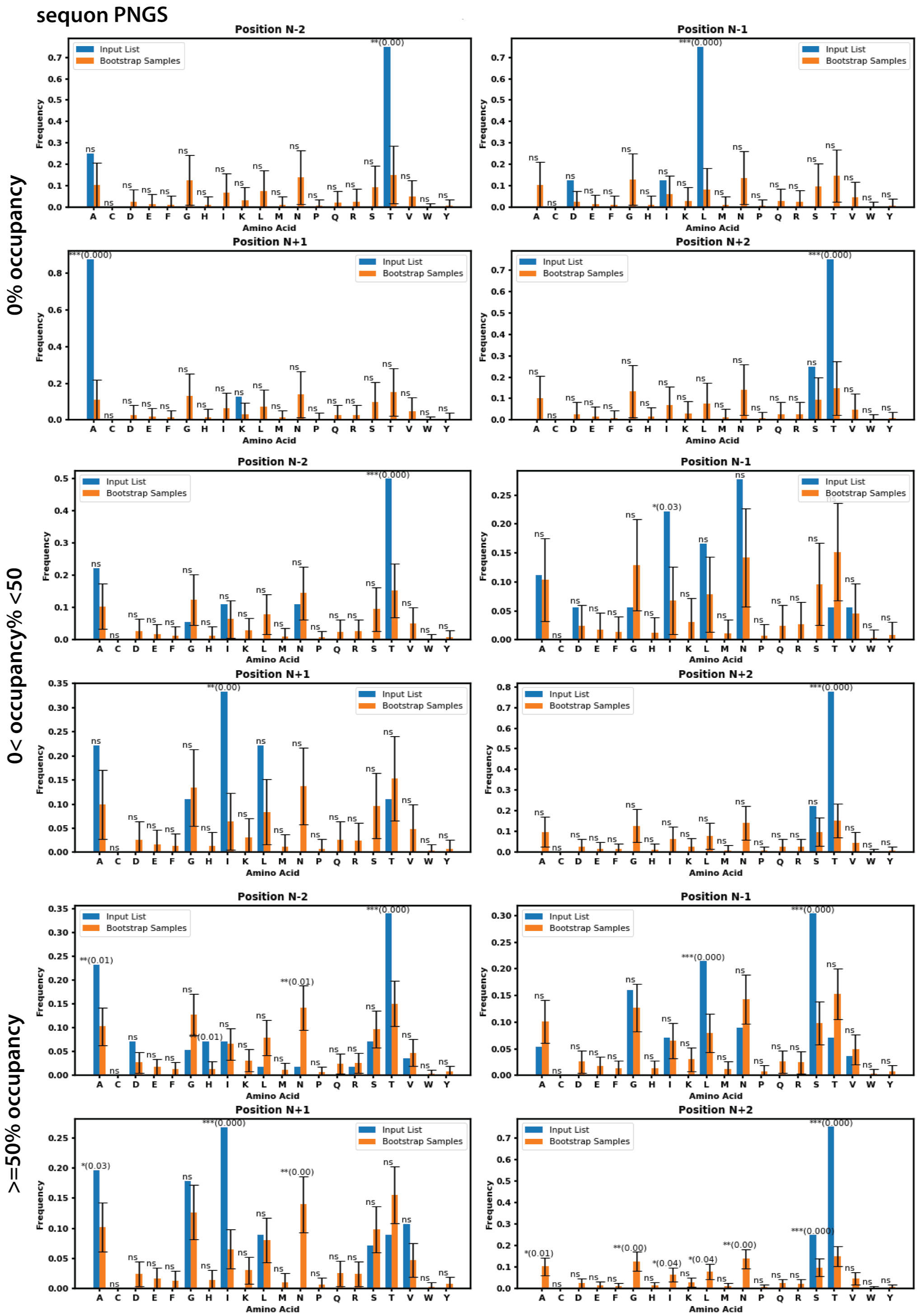

Supplement: S5 Fig — Statistical analysis of amino acid type frequencies at the 4 sites immediately upstream and downstream of each canonical sequon PNGS broken down by occupancy percentage as determined by mass-spectrometry. Blue bars are the average frequencies for the input residue list and orange bars are average frequencies for 1000 permutation samples with error bars and statistical significance measurements broken down by minor significance (*), median significance (**), and high significance (***) along with corresponding p-values. (TIF) [file ppat.1012241.s005.tif]

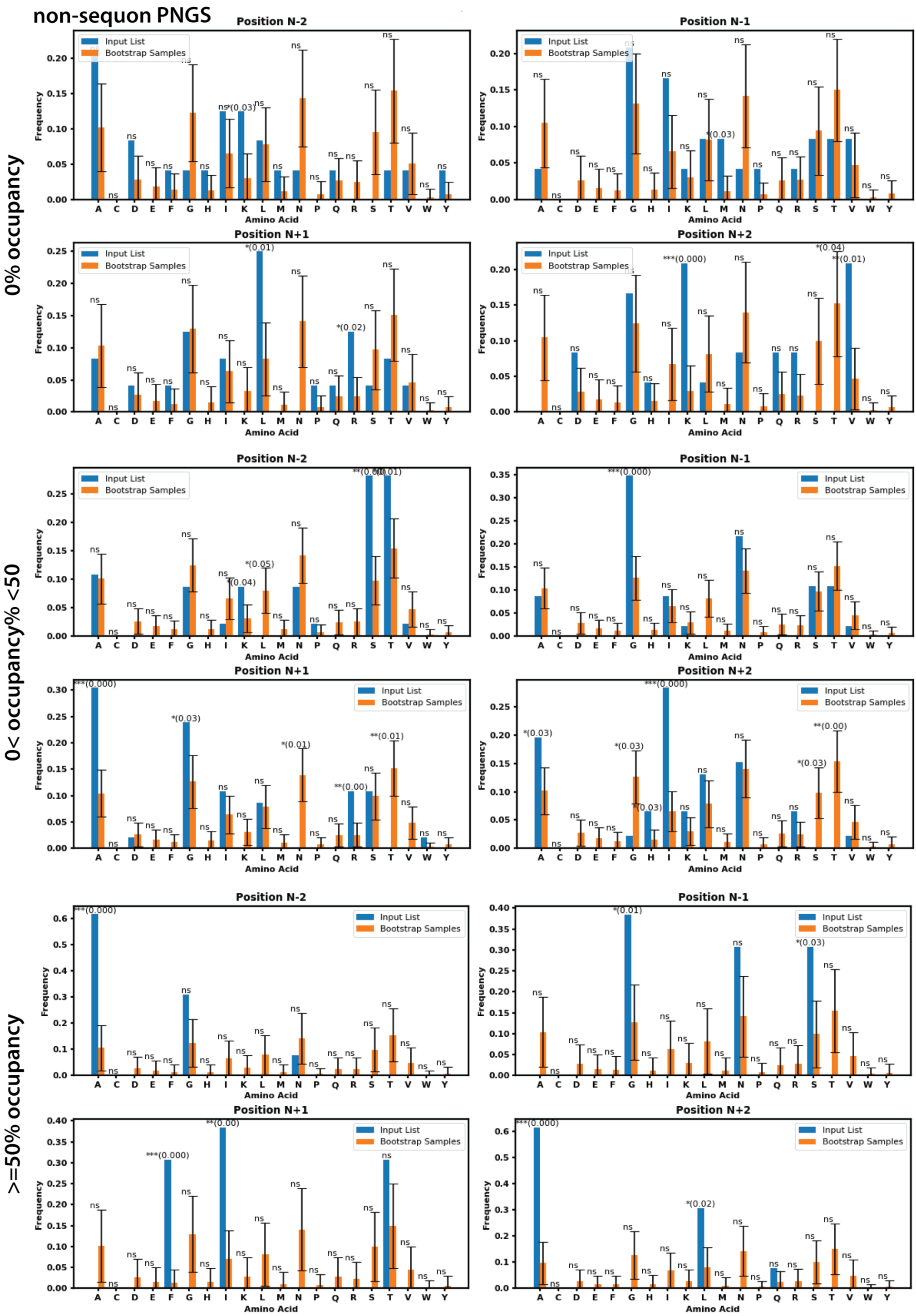

Supplement: S6 Fig — Same as in S5 Fig, but for all non-canonical sequon PNGS. (TIF) [file ppat.1012241.s006.tif]

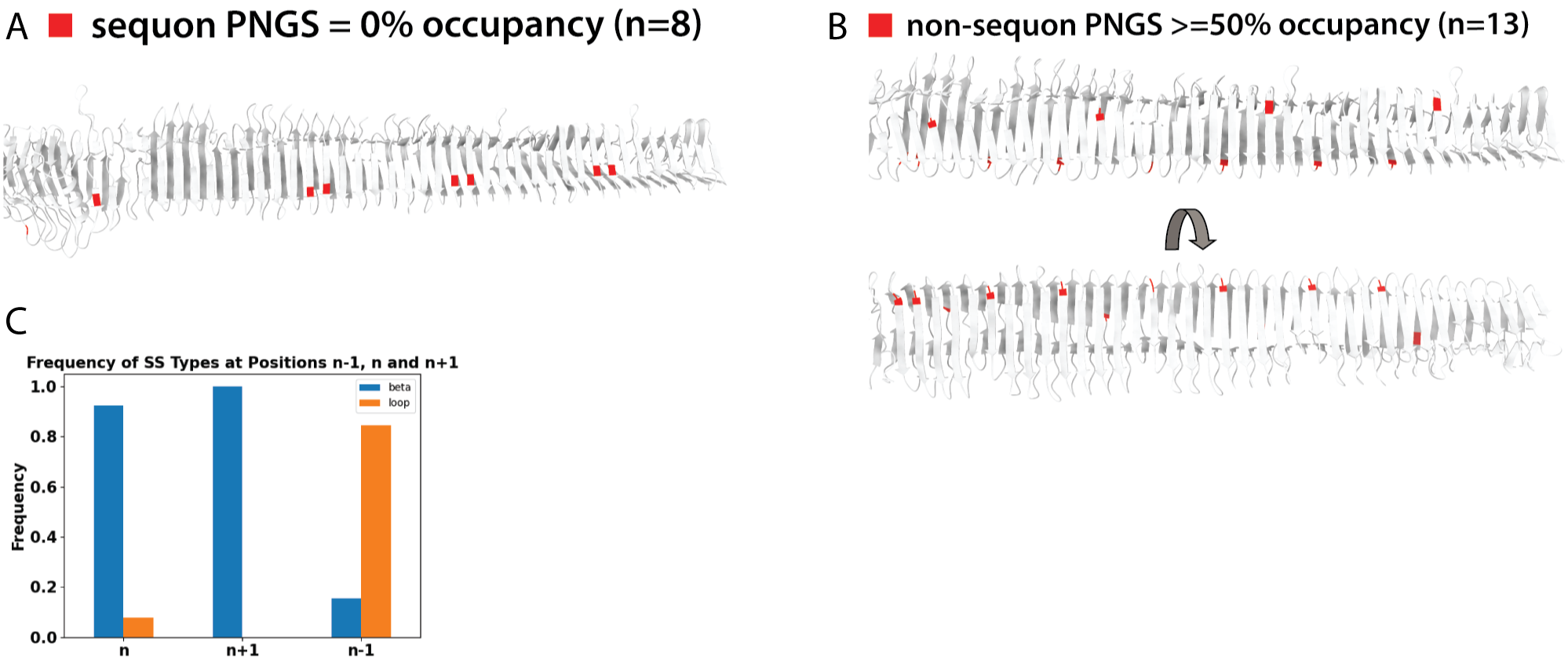

Supplement: S7 Fig — A. rEtpA structure with the 8 sequon PNGS sites with 0% occupancy colored red. B. rEtpA structure showing all non-sequon PNGS with ≥ 50% occupancy colored red. C. Bar plot showing the frequency of secondary structure types at the PNGS and the two residues immediately upstream and downstream of the site. (TIF) [file ppat.1012241.s007.tif]

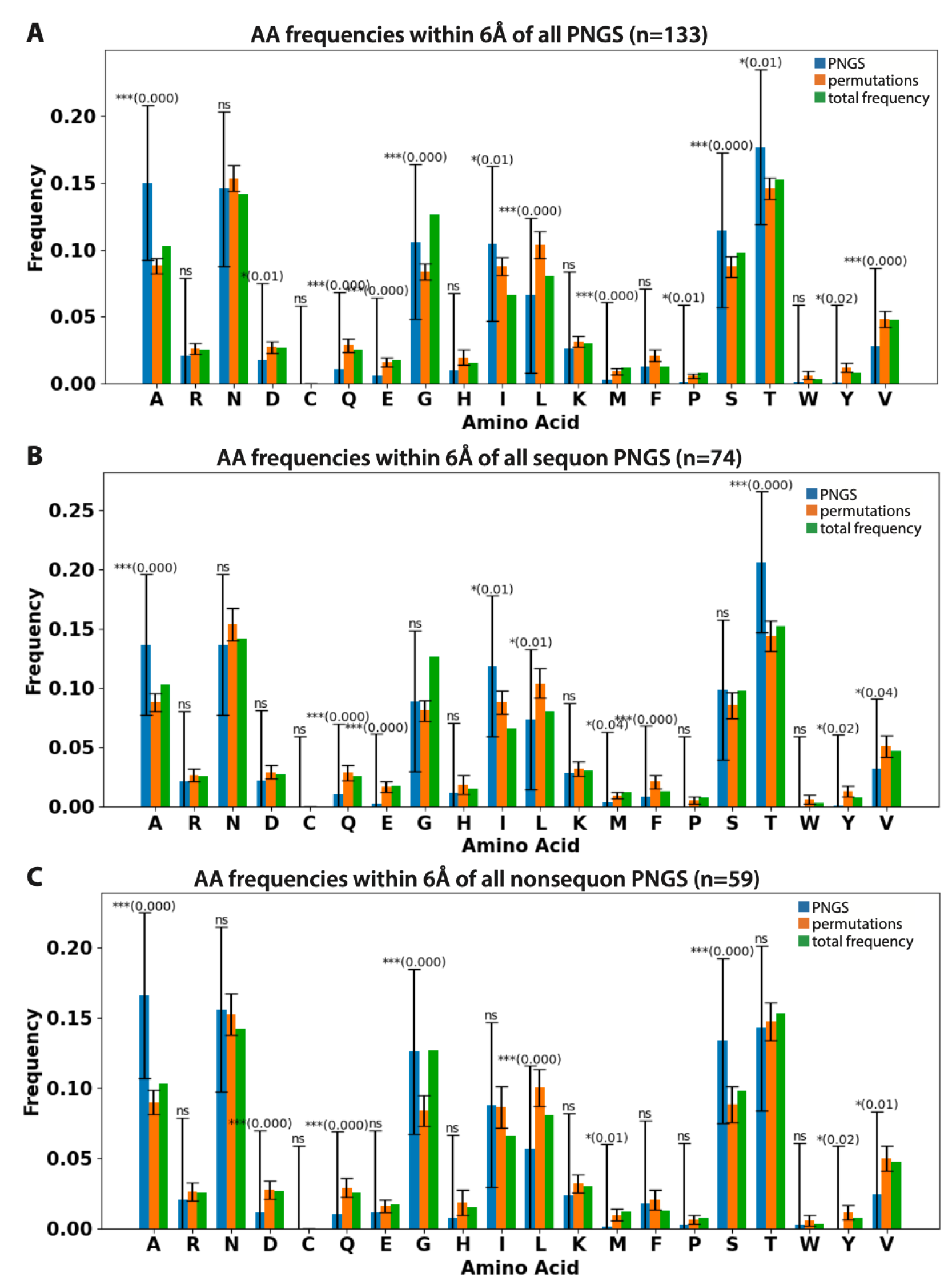

Supplement: S8 Fig — A. Bar plot showing the average frequency of each amino acid type within 6Å of all PNGS, canonical sequon PNGS (B), and non-canonical sequon PNGS (C). Blue bars are frequencies for the input list of residues, orange bars are the average frequencies across all 1000 permutation samples (with error bars and significance measures as described in S5 Fig), and green bars are the frequency of that amino acid within rEtpA. (TIF) [file ppat.1012241.s008.tif]

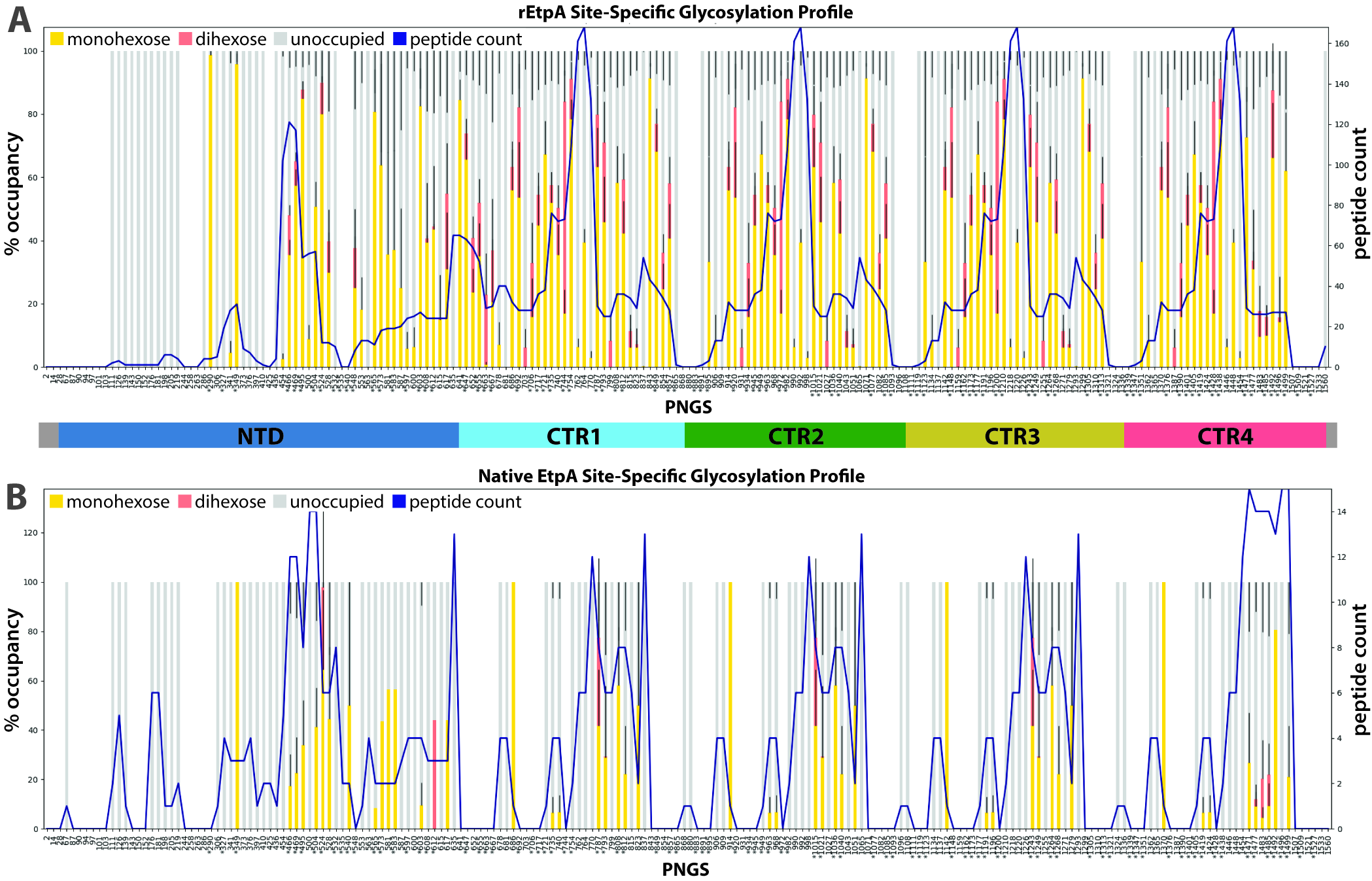

Supplement: S9 Fig — A. Glycosylation profile for rEtpA reproduced from Fig 3. B. Glycosylation profile of native EtpA from ETEC strain H10407. Left axis is % occupancy and right axis is peptide count. Note the much lower peptide counts for native EtpA. (TIF) [file ppat.1012241.s009.tif]

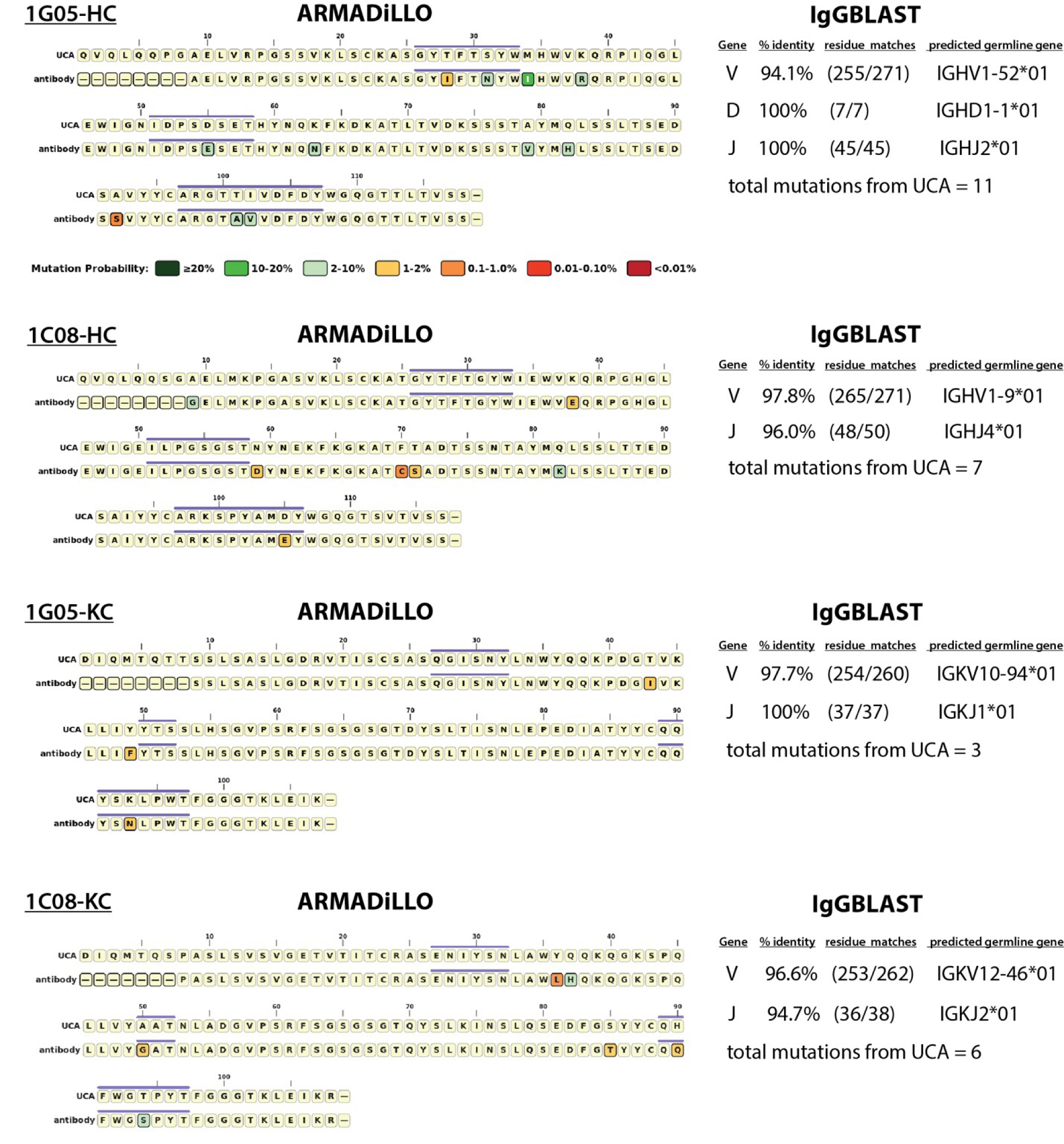

Supplement: S10 Fig — IgGBlast results summaries for 1C08 and 1G05 heavy chains (HC) and kappa chains (KC) showing the top match for V, D, and J mouse germline genes. Next to the summary tables are the percent identity to the top matching genes along with the gene names and the total number of somatic hypermutations (SHM) away from the predicted unmutated common ancestor (UCA). Also shown are output plots from the program ARMADILLO with SHM sites shown and scored by their probability, with red being the least probable and green being the most probable. (TIF) [file ppat.1012241.s010.tif]

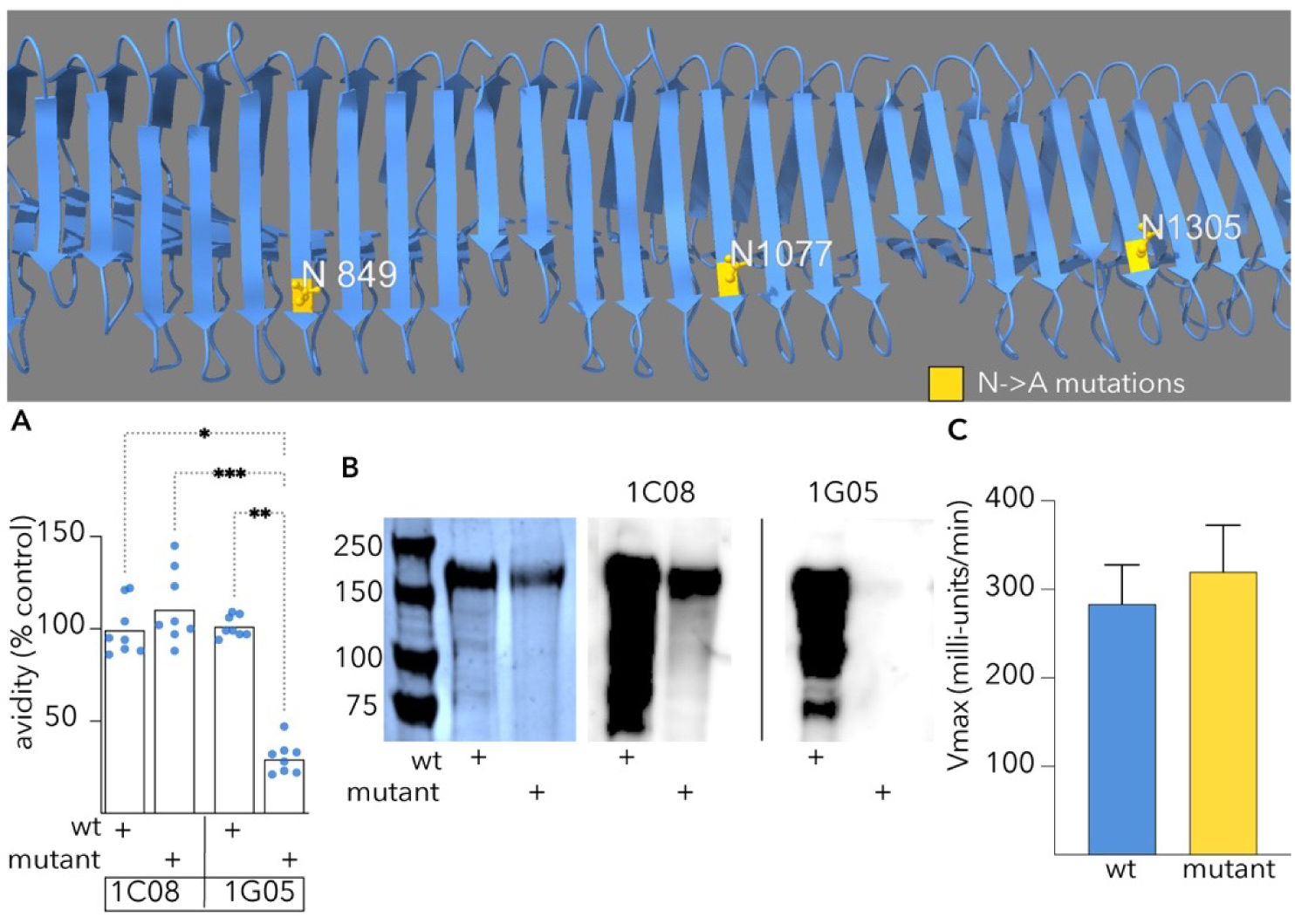

Supplement: S11 Fig — Figure at top depicts relative location of asparagine (N) to alanine (A) mutations to the putative 1G05 epitope. A. mAb 1G05 exhibits decreased avidity for recombinant mutant EtpA with N to A substitutions within C-terminal repeat region at positions 849, 1077, and 1305. Avidity indices (AI) were determined by kinetic ELISA with and without addition of 8 M urea as the chaotropic agent. AI (%) = (Vmax with urea)/(Vmax without urea) and expressed as % of the wild type recombinant protein. Comparisons by Kruskal-Wallis (n = 8 technical replicates/group from 2 independent experiments) *** = 0.0003, ** = 0.0037, * = 0.03. B. Immunoblot recognition of wild type and mutant protein by 1C08 and 1G05. PAGE image (left) indicates protein loading and MW markers. C. Blood group A binding by wild type and mutant protein in kinetic ELISA assay. (TIF) [file ppat.1012241.s011.tif]

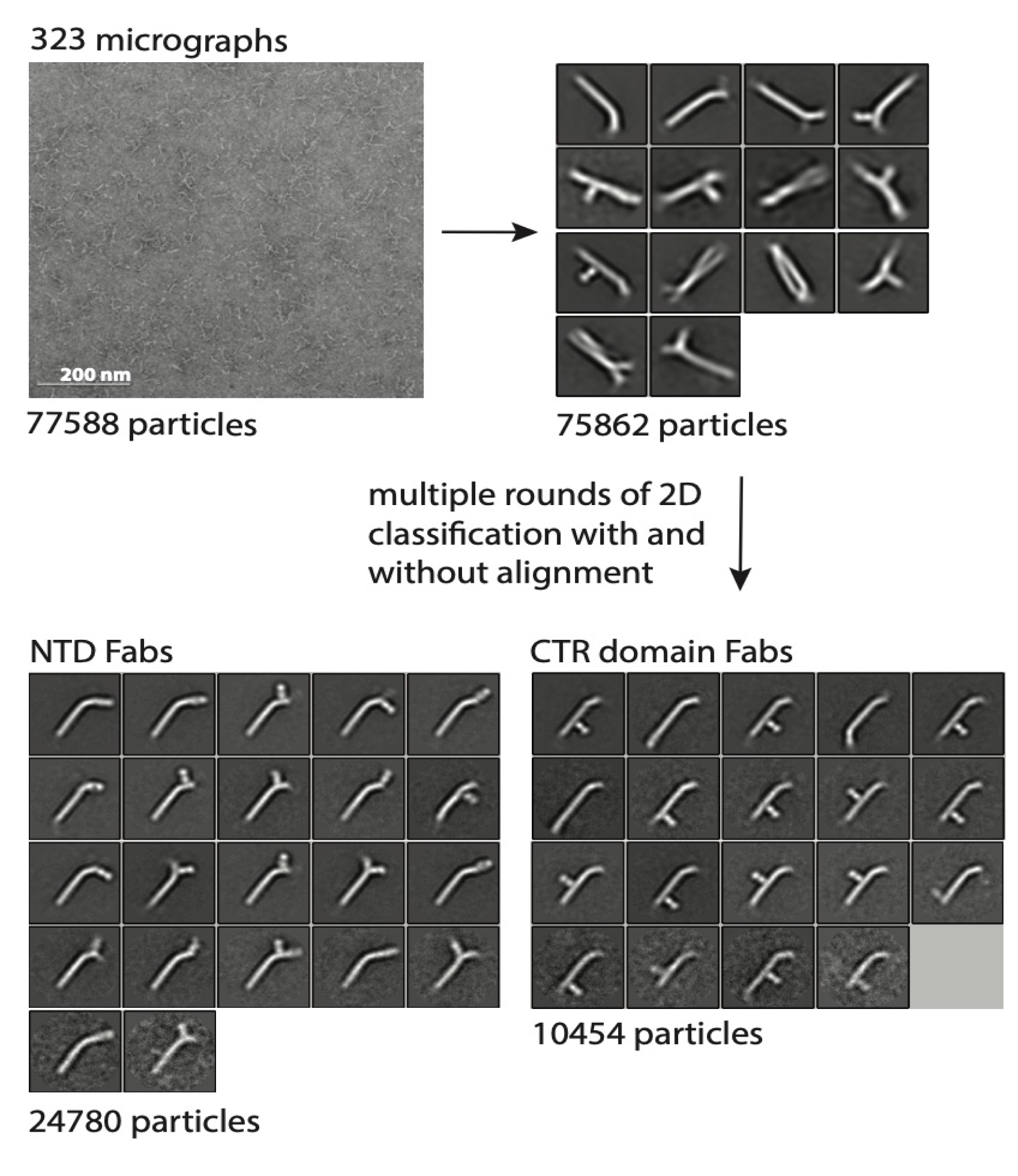

Supplement: S12 Fig — Simplified negative stain EMPEM data processing workflow including a representative micrograph, 2D class averages, and particle counts at each step. (TIF) [file ppat.1012241.s012.tif]

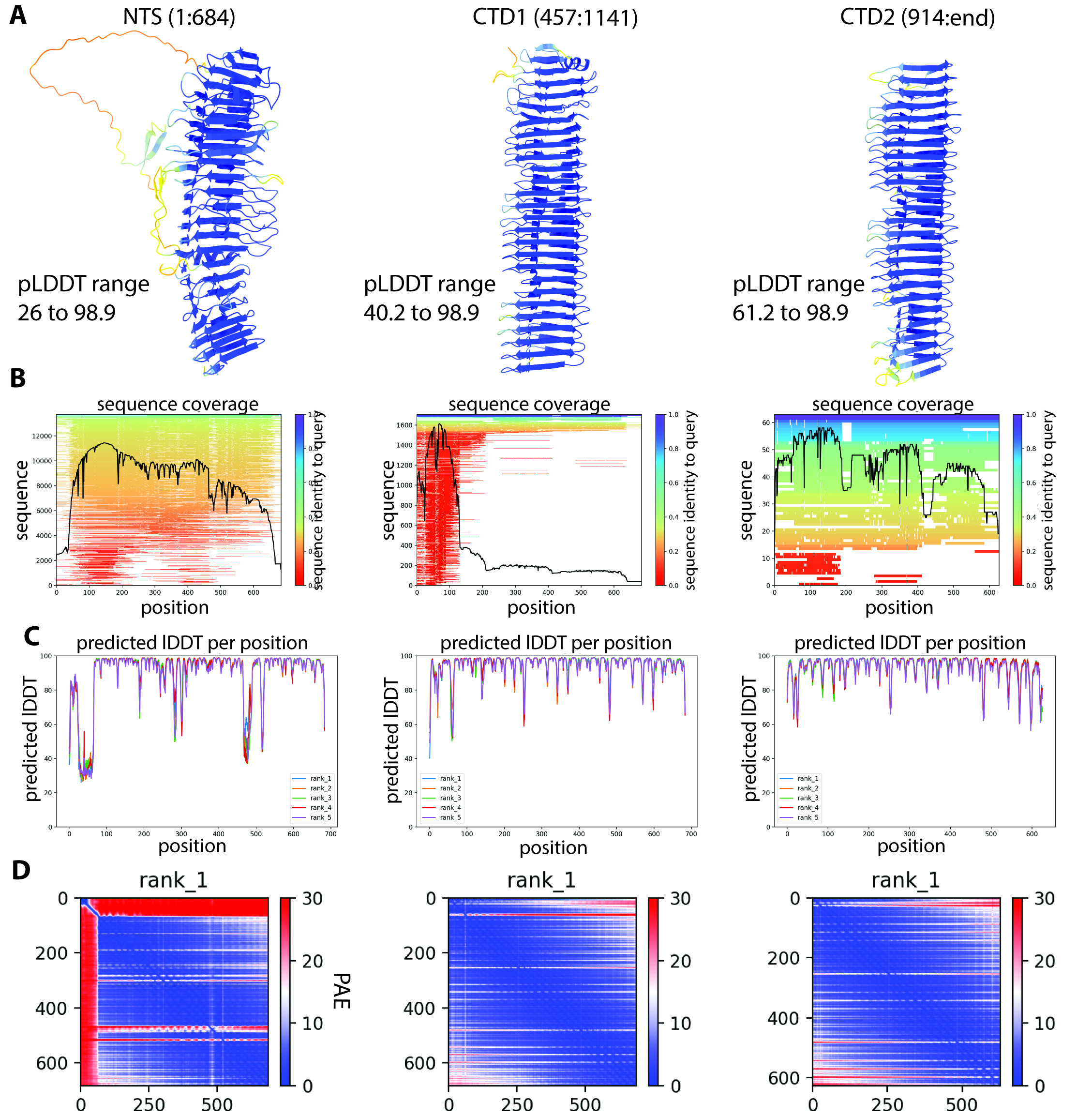

Supplement: S13 Fig — A. AlphaFold2 top ranking model colored by pLDDT score (blue = high). B. Per-residue sequence coverage and identity. C. Per-residue pLDDT scores. D. PAE (predicted aligned error) matrix. (TIF) [file ppat.1012241.s013.tif]
