# Supplementary material for: Repeat modules and N-linked glycans define structure and antigenicity of a critical enterotoxigenic E. coli adhesin
Source: PLoS Pathog. 2024 Sep 16;20(9):e1012241. doi: 10.1371/journal.ppat.1012241 (PMC11463764; doi:10.1371/journal.ppat.1012241)
Supplement: S1 Table — (PDF) [file ppat.1012241.s014.pdf]

S1 table strains and plasmids			
strain	description/genotype	reference	
Top10	F–mcrA (mrr-hsdRMS-mcrBC) 80lacZM15 lacX74 recA1 araD139 (ara-leu)7697 galU galK – rpsL(StrR) endA1 nupG	Invitrogen	
jf1696	Top10(pJL017/pJL030), AmpR, CmR	[1, 2]	
jf2826	LMG194 *fliC*::KmR	[1]	
jf3013	Top10(pQL211/pJL030), AmpR, CmR	this study	
jf3099	H10407 *fliC*::KmR	[1]	
jf5090	Top10(pMH4/pJL030) AmpR, CmR	this study	
jf5381	jf2826(pJL030)		
jf5500	jf5381(pJL030/pTV005) AmpR, CmR	this study	
H10407	wild type ETEC strain *etpBAC* serotype O78:H11, LT/STh/STp	[3, 4]	
plasmid	description	reference(s)	addgene number
pJL030	pACYC184 based etpC expression plasmid CmR	[1, 5]	53533
pJL017	etpBA cloned into pBAD/Myc- His A*, with etpA in-frame with myc and 6His coding regions	[35]	53532
pJMF1028	linker scanning mutant of pJL017 with transprimer insertion at 3258 of *etpA*	[1]	
pMH4	EtpA-amplified from pJMF1028, encodes EtpA (AA 1-1086)	this study	
pQL211	N-terminal region EtpA subclone (AA 1-607) generated with primers jf02214.3/jf022114.4 cloned in-frame with myc and 6His coding regions of pBAD/Myc- His A.	[6]	
pTV005	Mutant EtpA expression plasmid bearing N849A, N1077A, and N1305A mutations	this study	
KmR=kanamycin resistant; AmpR=beta-lactamase/ampicillin resistant; CmR=chloramphenicol resistant.			

references

1. Roy K, Hilliard GM, Hamilton DJ, Luo J, Ostmann MM, Fleckenstein JM. Enterotoxigenic Escherichia coli EtpA mediates adhesion between flagella and host cells. Nature. 2009;457(7229):594-8. Epub 2008/12/09. doi: 10.1038/nature07568. PubMed PMID: 19060885; PubMed Central PMCID: PMC2646463.
2. Roy K, Hamilton D, Allen KP, Randolph MP, Fleckenstein JM. The EtpA exoprotein of enterotoxigenic Escherichia coli promotes intestinal colonization and is a protective antigen in an experimental model of murine infection. Infection and immunity. 2008;76(5):2106-12. Epub 2008/02/21. doi: IAI.01304-07 [pii]
10.1128/IAI.01304-07. PubMed PMID: 18285493; PubMed Central PMCID: PMC2346670.
3. Evans DJ, Jr., Evans DG. Three characteristics associated with enterotoxigenic Escherichia coli isolated from man. Infection and immunity. 1973;8(3):322-8. doi: DOI: 10.1128/iai.8.3.322-328.1973. PubMed PMID: 4581006; PubMed Central PMCID: PMCPMC422851.
4. Fleckenstein JM, Roy K, Fischer JF, Burkitt M. Identification of a two-partner secretion locus of enterotoxigenic Escherichia coli. Infection and immunity. 2006;74(4):2245-58. PubMed PMID: 16552055.
5. Fleckenstein JM, Roy K. Purification of recombinant high molecular weight two-partner secretion proteins from Escherichia coli. Nat Protoc. 2009;4(7):1083-92. Epub 2009/08/27. doi: 10.1038/nprot.2009.87. PubMed PMID: 19707189.
6. Chakraborty S, Randall A, Vickers TJ, Molina D, Harro CD, DeNearing B, et al. Interrogation of a live-attenuated enterotoxigenic Escherichia coli vaccine highlights features unique to wild-type infection. NPJ Vaccines. 2019;4:37. Epub 2019/09/05. doi: 10.1038/s41541-019-0131-7. PubMed PMID: 31482013; PubMed Central PMCID: PMCPMC6713706.
