# Supplementary material for: Repeat modules and N-linked glycans define structure and antigenicity of a critical enterotoxigenic E. coli adhesin
Source: PLoS Pathog. 2024 Sep 16;20(9):e1012241. doi: 10.1371/journal.ppat.1012241 (PMC11463764; doi:10.1371/journal.ppat.1012241)
Supplement: S2 Table — (PDF) [file ppat.1012241.s015.pdf]

**S2 table primers**	
primer	sequence 5'->3'
jf051716.1	GAGGAATTAACC**ATGG**TGGTGAAATTCATGTCAG
jf082718.1	TTGTTCTAGAAAGC**TT**CCCAACTTTATTGTGTTTAAACA
jf022014.3	ACTTCAAACCGTGTGAATATATCTATTAATAAGCTTGGGCCCGAAC
jf022014.4	GTTCGGGCCCAAGCTTATTAATAGATATATTCACACGGTTTGAAGT
pBAD reverse	GATTTAATCTGTATCAGG
Regions in bold correspond to 5' end of *etpB* sequence for jf051716.1, and to 3' end of the GPS4 Tn7L transprimer sequence for jf082718.1. Underlined nucleotides represent *NcoI* and *HindIII* sites, respectively.	
