# Supplementary material for: Repeat modules and N-linked glycans define structure and antigenicity of a critical enterotoxigenic E. coli adhesin
Source: PLoS Pathog. 2024 Sep 16;20(9):e1012241. doi: 10.1371/journal.ppat.1012241 (PMC11463764; doi:10.1371/journal.ppat.1012241)
Supplement: S3 Table — (PDF) [file ppat.1012241.s016.pdf]

S3. Table Cryo-EM data collection, refinement, and validation statistics

	rEtpA:1C08 Fab (EMDB-xxxx) (PDB xxxx)	rEtpA:1G05 Fab (EMDB-xxxx) (PDB xxxx)
**Data collection and processing**		
Magnification	97,0000	97,0000
Voltage (kV)	200	200
Electron exposure (e–/Å^2)	47	47
Defocus range (μm)	~-0.25:-2.0um	~-0.4:-2.0um
Pixel size (Å)	0.725Å	0.725Å
Symmetry imposed	none	none
Initial particle images (no.)	4,178,645	1,651,766
Final particle images (no.)	215,360	52,107
Map resolution (Å)<br>FSC threshold	3.34Å<br>0.143	3.97Å<br>0.143
Map resolution range (Å)	~3-5Å	~3.5-5Å
Map sharpening B factor (Å^2)	110.5	380.4
**Refinement**		
**Initial model used (PDB code)**	**AlphaFold2 and SABPred predictions**	**AlphaFold2 and SABPred predictions**
Model composition<br>Non-hydrogen atoms<br>Protein residues<br>Ligands	 9416<br>1260<br>39 BGC	 9444<br>1260<br>38 BGC
R.m.s. deviations<br>Bond lengths (Å)<br>Bond angles (°)	 0.011<br>1.318	 0.01<br>1.03
Validation<br>MolProbity score<br>Clashscore<br>Poor rotamers (%)<br>CaBLAM outliers (%)	 2.03<br>14.87<br>0.51<br>2.8	 2.02<br>15.05<br>0.41<br>3.53
Ramachandran plot<br>Favored (%)<br>Allowed (%)<br>Disallowed (%)	 94.90<br>4.63<br>0.48	 95.14<br>4.63<br>0.24
EMRinger score	3.6	2.7
