# Supplementary material for: Repeat modules and N-linked glycans define structure and antigenicity of a critical enterotoxigenic E. coli adhesin
Source: PLoS Pathog. 2024 Sep 16;20(9):e1012241. doi: 10.1371/journal.ppat.1012241 (PMC11463764; doi:10.1371/journal.ppat.1012241)
Supplement: Attachment — Submitted filename: PPATHOGENS-D-24-09432.2_response.pdf [file ppat.1012241.s017.pdf]

Washington University in St. Louis

SCHOOL OF MEDICINE

Reviewer's Responses to Questions

Part I - Summary

Please use this section to discuss strengths/weaknesses of study, novelty/significance, general execution and scholarship.

Reviewer #1: This manuscript on the structure, antigenicity, and potential of the secreted EtpA protein as a vaccine candidate against diarrheal disease caused by ETEC includes multiple experiments that advance the field.

Reviewer #2: This study is an important advancement in the understanding of ETEC EtpA structure and immunogenicity, especially regarding the roles of N-linked glycosylation, an increasingly recognized post-translational modification of a growing number of adhesins in a variety of gram-negative bacteria. The use of cryo-EM on EtpA-Fabs (from both monoclonal and polyclonal mouse and human sources) complexes revealed the location and specific residues involved in key EtpA-antibody interactions and enabled structural characterization of glycosylated EtpA. Mass spectrometry approaches uncovered the breadth of glycosylation of EtpA and revealed the impact of specific glycosylation sites on antibody binding were revealed.

Reviewer #3: The authors of this study use a combination of sophisticated structural biology, immunology, mass spectrometry, molecular biology, and ligand binding techniques in the service of investigating the highly conserved EtpA adhesin from enterotoxigenic E. coli. Although the crystal structure of EtpA had previously been solved, their cryo-EM structure in complex with two monoclonal antibodies, one that does and one that does not inhibit binding to Group A blood antigens on solid surfaces and tissue culture cells, provides new insights into the role of surface sugars in binding and suggest important epitopes that may be targeted with protective antibodies. The use of polyclonal serum from infected humans to further characterize binding sites using negative-stained EM and 2D particle analysis adds considerable confirmatory data of particular relevance to human health. The methods are well described, the results are convincing, and the conclusions are important and well-justified.

We appreciate these comments from the reviewers. We only wish to point out that like most other two-partner secretion proteins, only the N-terminal secretion domain of EtpA had been crystalized and structurally characterized in the earlier paper[1]. Therefore, the C-terminal repeat structure included in the present manuscript is new.

Part II – Major Issues: Key Experiments Required for Acceptance

Please use this section to detail the key new experiments or modifications of existing experiments that should be absolutely required to validate study conclusions.

Generally, there should be no more than 3 such required experiments or major modifications for a "Major Revision" recommendation. If more than 3 experiments are necessary to validate the study conclusions, then you are encouraged to recommend "Reject".

Reviewer #1: (No Response)

Reviewer #2: Major points:
• Lines 290-293. The statement that there is “close agreement” between the rEtpA and native EtpA glycosylation patterns is difficult to believe given the comparison shown in Supp. Fig 7. These glycosylation profiles illustrated in Supp. Fig 7 look markedly different. Were other digestion enzymes (e.g, chymotrypsin) attempted to increase coverage of the protein and/or a larger scale purification attempted to increase yield? The apparent differences in the profiles need to be addressed.

• The recombinant expression system involving production of EptC from one plasmid and induction of EptA from another plasmid will most likely result in a scenario in which the molar ratio of EptC:EptA in the recombinant system differs significantly from the molar ratio of EptC:EptA in the native strain. This difference in molar ratio is likely to have significant impact on the glycosylation profiles in the two systems, a limitation should be acknowledged and addressed in the Discussion.

The reviewer raises important points with respect to potential differences in the glycosylation patterns of the wild type EtpA adhesin and the recombinant protein. We embarked on these studies precisely because of potential artifacts arising from the two-plasmid expression system employed in production of the recombinant antigen, and the concern that rEtpA may not faithfully recapitulate the glycosylation of the native antigen. Despite attempting multiple approaches to enhance the yield of the native protein, yields were typically limited relative to that of recombinant EtpA. The differences observed in figure 7 arise primarily from not being able to generate a sufficient number of peptide fragments during mass-spec for accurate mapping of the native protein glycosylation. Nevertheless, for those PNGS with sufficient peptide coverage, we do see agreement with the recombinant protein. More importantly, the defining feature of heterogeneous occupancy is recapitulated in the WT protein, as we observe mixtures of monohexose, dihexose, and no glycan at most well-sampled sites. We have amended this section of the manuscript and have addressed this issue in the revised discussion.

Reviewer #3: None noted

Part III – Minor Issues: Editorial and Data Presentation Modifications
Please use this section for editorial suggestions as well as relatively minor modifications of existing data that would enhance clarity.

Reviewer #1: The minor comments below need to be addressed. In addition, the large number of typographical errors in the submitted manuscript was distracting.

L 64 The structure presented is not at atomic structure, and the text should not state that it is.
removed "atomic"
L 91 is TpsA a synonym for EtpA? -- or is TpsA a class of proteins as implied on L 140? Your definition (L 88) seems to imply that Tps is the class (not TpsA), so your nomenclature is confusing
TpsA and TpsB refer to the generic terms for the secreted two partner exoprotein and membrane protein of TPS systems, respectively. We have amended this paragraph to clarify.

Fig 1B - what is 'membrane'? images have weird edges.
The (pseudocolored) "membrane" images were generated after staining plasma membranes with CellMask (the figure legend has been amended to reflect this). The weirdness the reviewer may be referring to are the yz and xz planes from the original confocal images which are difficult to see at this magnification.
Fig 2 what portion of A is shown in B? what is the relative orientation of panels A and B? relative orientation of A and C? panel D, NTD vs NTS; NTD is only used here and one other place in the manuscript, and therefore it is confusing. How does this relate to the term N-terminal (TPS) domain, referred to in the legend of Fig 2B. -- NTD=NTS plus 65 N-term residues?

Panel B shows the isolated N-terminal domain, which is stated in the figure legend. The residue numbers corresponding to the NTD are designated in the gene diagram in panel A.

The orientation of panel B is a "top-down" view of the NTD. The figure has been updated to make this clearer as has the figure legend. Panel C is also a top-down view of the NTD, but just oriented perpendicular to panel B to better fit the space. We have also updated the legend to point this out.

NTD=NTS. We have removed any reference to the NTD and changed it to NTS in the figure to match the rest of the text.

L 180-181 -- need to define an "extra-helical insert", because it appears that CTR4 has them too; you have a minimum length?

The appearance of longer loops within CTR4 is artifactual due to the sub-optimal beta-sheet geometry of the last few beta-strands. The molecular visualization software used to make these figures (Chimera) will only draw beta-sheet ribbons if the geometry matches proper beta-sheet geometry. Because the resolution of the cryo-EM map attenuates at it approaches the C-terminus, the model refinement step resulted in non-ideal beta-sheet geometry in these last few strand, which gives the appears of there being longer loops, when in fact, they are identical to the same inter-strand loops in the other 3 CTRs.

L 187-188 "potentially enhancing flexibility between the two domains". if this is true, then the

cryoEM map should have limited resolution in this area, and this can be quantitatively shown. If the data don't show this, then remove the statement from results, and put it in discussion

Flexibility between the NTS and CTR domains was observed during cryo-EM data processing, however, 3-D classification was used to reduce the effects of this variability on the final reconstruction to achieve more uniform resolution throughout the entire map.

L 199 - H2 is not shown in Fig 2, so text is unclear

H2 was labeled as "a-helix 2" in the figure. It has now been updated to H2.

Fig 3 - is it the graphed line that is the peptide count? if so, the legend on the figure and in its text should indicate this.

The figure legend was updated to indicate that the right axis and blue line are peptide count

This correct. We have amended figures 3 and supplemental figure 7 to clarify this.
panel D cross-section, label the faces. panel E - explain the BSA column in the table; a discussion of this result should be in the text of the manuscript, since this is such a large number compared to the rEtpA results
Figure 3 legend amended to define BSA as "Buried Surface Area" and the faces of the beta-helix have now been labeled
L 334 -define EMPEM
Amended the subsection heading to define Electron Microscopy Polyclonal Epitope Mapping (EMPEM).
L 395-396 - finding 10 discrete polyclonal antibodies from the 600 amino acid NTD and 10 from the 900 amino acid CTR protein does not coincide with the statement that 'rEtpA possesses a large immunogenic surface'. Polyclonal antibodies are often seen decorating the entire surface of a protein, appearing as a fuzzy coat; the data do not support your statement.

We believe that the statement that EtpA presents a large immunogenic surface is accurate as written in that multiple unique epitopes within both N and C-terminal domains are discernable. EMPEM inherently has a limitation in that antibodies bound with the antigen are isolated by size exclusion chromatography, meaning that lower affinity antibodies are not necessarily retained. Those identified by EMPEM here are unique based on their location and angle of approach to the antigen, but the depiction of 20 distinct epitopes illustrated in figure 5 is not exhaustive and it likely that other epitopes are targeted. In other examples of EMPEM data, for instance in the context of viral antigens such as Influenza HA, multiple antibodies can often been seen bound to the same antigen, which may be what the reviewer is referring to when they say "fuzzy coat." While we did not observe 2-D class averages with more than a few Abs bound at once, this does not exclude the possibility that multiple Abs can target the same antigen simultaneously. The data processing approach we took with this data involved more extensive 2-D sorting than is typically performed. If less extensive sorting is performed, the averaging process can often give the artificial appearance of multiple Abs bound to the same antigen.

Reviewer #2: Minor points:
• Figure 1G: The meaning of the blue vs open circle data points is unclear.

Legend for 1G amended to reflect that these represent experimental replicates.

• Figure 3D: The text in lines 262-263 indicates that panel 3D is a cryo-EM map, but the figure legend indicates that it is a structure with modeled N-linked glucose residues added. Please clarify.

This line has been amended to indicate that panel D shows the refined atomic model, not the cryo-EM map. The statement was meant to convey that a hexose residue was modeled at all sites where density was observed in the cryo-EM map for hexose residues.

• Many of the figures are very small, with labels and annotation that will be very difficult to read in the published paper. Examples include Fig 1 (especially 1A), Fig 3, Suppl Fig 2 (especially 2B), Suppl Fig 3 (especially Fig 3B), Suppl Fig 4 (especially Fig 4D), Suppl Fig 5, Suppl Fig 11 (especially Fig 11B, 11C, and 11D).
Agreed. We will fix these prior to production where possible.

In figure 3A, however, it is not possible to label the individual PNGS in the x-axis with larger font while still being able to fit all the labels on the figure. This is unfortunate, but readers will be able to zoom into the high-resolution images if they need to find specific PNGS.

For supplemental Figure 5, in order to make each panel large enough to properly read, we had to split the figure into 3 parts which we have now labeled SI Figure 5, 5 cont., and 5 cont. cont..
Reviewer #3: Humans are the only species that can volunteer, so the authors should search for every use of "human volunteers" and eliminate this redundancy.
True. We certainly appreciate this keen insight of the reviewer, and we have eliminated all references to “human” volunteers.
Severity appears twice in the sentence spanning lines 38-40.
Corrected. Thank you.
The word "to" should be deleted from line 70.
We have revised this section of the author summary.

In line 253 "that" should be "than".
Corrected in revision

Where is the cartoon representation to which line 844 refers?
Figure 5 legend was amended to delete this.

----

references
1. Ntui, C.M., Fleckenstein, J.M., and Schubert, W.D. (2023). Structural and biophysical characterization of the secreted, beta-helical adhesin EtpA of Enterotoxigenic Escherichia coli. PLoS One 18, e0287100. 10.1371/journal.pone.0287100.
